# Flow Interactions Between Low Aspect Ratio Hydrofoils in In-line and Staggered Arrangements

**DOI:** 10.3390/biomimetics5020013

**Published:** 2020-03-31

**Authors:** Melike Kurt, Azar Eslam Panah, Keith W. Moored

**Affiliations:** 1 Mechanical Engineering and Mechanics, Lehigh University, Bethlehem, PA 18015, USA; kmoored@lehigh.edu; 2 Mechanical Engineering, Division of Engineering, Business and Computing, Pennsylvania State University at Berks, Reading, PA 19610, USA; aue10@psu.edu

**Keywords:** collective swimming, bio-inspired propulsion, fluid-structure interactions, propulsive performance, unsteady aerodynamics, fish schooling

## Abstract

Many species of fish gather in dense collectives or schools where there are significant flow interactions from their shed wakes. Commonly, these swimmers shed a classic reverse von Kármán wake, however, schooling eels produce a bifurcated wake topology with two vortex rings shed per oscillation cycle. To examine the schooling interactions of a hydrofoil with a bifurcated wake topology, we present tomographic particle image velocimetry (tomo PIV) measurements of the flow interactions and direct force measurements of the performance of two low-aspect-ratio hydrofoils (AR=0.5) in an in-line and a staggered arrangement. Surprisingly, when the leader and follower are interacting in either arrangement there are only minor alterations to the flowfields beyond the superposition of the flowfields produced by the isolated leader and follower. Motivated by this finding, Garrick’s linear theory, a linear unsteady hydrofoil theory based on a potential flow assumption, was adapted to predict the lift and thrust performance of the follower. Here, the follower hydrofoil interacting with the leader’s wake is considered as the superposition of an isolated pitching foil with a time-varying cross-stream velocity derived from the wake flow measurements of the isolated leader. Linear theory predictions accurately capture the time-averaged lift force and some of the major peaks in thrust derived from the follower interacting with the leader’s wake in a staggered arrangement. The thrust peaks that are not predicted by linear theory are likely driven by spatial variations in the flowfield acting on the follower or nonlinear flow interactions; neither of which are accounted for in the simple theory. This suggests that unsteady potential flow theory that *does* account for spatial variations in the flowfield acting on a hydrofoil can provide a relatively simple framework to understand and model the flow interactions that occur in schooling fish. Additionally, schooling eels can derive thrust and efficiency increases of 63-80% in either a in-line or a staggered arrangement where the follower is between two branched momentum jets or with one momentum jet branch directly impinging on it, respectively.

## 1. Introduction

**Figure 1:**
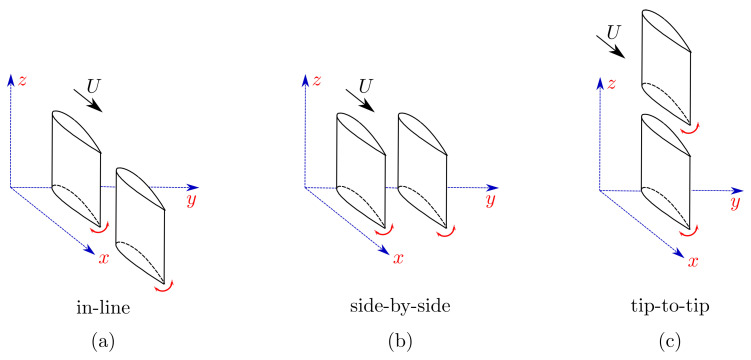
Schematics of the canonical arrangements; in-line (**a**), side-by-side (**b**), and tip-to-tip (**c**).

In nature, many aquatic animals propel themselves by oscillating their bodies, fins, and tails, and often times they are known to aggregate in collectives or schools. Locomotion within schools have inspired many studies that link this behavior to social reasons [1], protection against predators [2], and even to a reduction in energy expenditure [3]. The close proximity of individuals within schools inevitably alters the surrounding fluid flow, and consequently changes the force production and energy expenditure for individual swimmers, and potentially for the whole collective. In fact, there have been extensive research efforts to understand the force production and energetics of animals within collectives estimating, for example, that fish schools can achieve a 15% thrust enhancement [3], and pelicans in a V-formation can save 11–14% of their energy [4]. In a similar way, flow interactions between synchronized dorsal and caudal fins on an isolated fish can improve the thrust performance and reduce its energy expenditure [5,6]. Several studies have demonstrated that the synchronization and spatial location of individuals within collectives is of prime importance in determining their force production and energetics [3,7,8,9]. These highly three-dimensional spatial configurations found within collectives can be decomposed into canonical in-line, side-by-side, or tip-to-tip arrangements as presented in Figure 1. To date, these interactions have mostly been studied for propulsors in in-line arrangements [10,11,12,13,14,15,16,17,18,19], although a few efforts have been made to understand interactions in side-by-side arrangements [20,21,22,23], as well as staggered arrangements [24,25,26,27]. Here, our focus is on the propulsive performance and flow interactions in *in-line*, and *staggered* arrangements.

**Figure 2:**
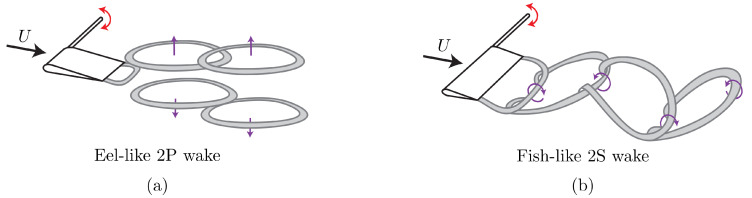
Schematics of two different wake topologies behind a finite-span pitching hydrofoil where (**a**) vortex rings bifurcate away from each other in the cross-stream direction as they advect downstream, commonly seen in eel-like swimming, and (**b**) interconnected vortex rings advect downstream, commonly seen in fish-like swimming.

Including hydrodynamic interactions in a model of a fish school is a challenging task without simplifications made with theoretical approximations [28]. Our current understanding of flow interactions and energetics within fish schools is mostly based on two-dimensional flow analyses [10,11,12,13,14,20,21,24,25,29,30], but there are far fewer three-dimensional studies focusing on these interactions that employ finite-span wings or hydrofoils, or fish-like bodies [15,19,22,25,31,32]. Amongst these studies, some consider the interaction of an oscillatory body with a classic drag-producing von Kármán street [30,33], and the others consider an interaction with a thrust-producing reverse von Kármán street [12,19,20]. However, some fish schools are composed of swimmers that do not produce classic reverse von Kármán wakes such as eels. Eels are known to school [34] and they shed a series of vortex rings that propagate away from each other (Figure 2a) that is sometimes described as a bifurcating wake [35,36]. In contrast, a three-dimensional reverse von Kármán wake takes the form of a series of interconnected vortex rings as presented in Figure 2b. In a numerical study conducted with eel-like swimmers and a deep reinforcement learning algorithm, Verma et al. [32] reported that a swimmer interacting with neighboring swimmers’ wakes often place themselves to harvest the energy of the shed vortex rings from a leader in order to maximize efficiency. Therefore, the optimal spatial arrangement that promotes high swimming efficiency can greatly vary depending on the wake topology produced by a leader.

Bifurcating wakes can occur when propulsors are operating at high Strouhal numbers (St≥0.4) and/or for low aspect ratios even if the kinematics and propulsor shape are not eel-like. For example, Buchholz and Smits [36] studied rigid, rectangular panels at different aspect ratios, undergoing pitching oscillations by using dye visualization and particle image velocimetry (PIV). In the wake of AR=0.54 panels operating at St≥0.4, they visualized two vortex rings bifurcating in opposite directions from the symmetry line. Similarly, Dong et al. [37] conducted a series of numerical simulations to investigate the effect of aspect ratio on the wake topology and propulsive performance of thin ellipsoidal flapping hydrofoils for Strouhal numbers up to 1.2, and they found a similar wake topology for an AR=1.27 hydrofoil operating at St≥0.4. Yet again, a bifurcating wake is observed for combined heaving and pitching hydrofoils of AR=3 at St=0.4 [38], and square pitching panels (AR=1) at St=0.4 [39].

Motivated by these studies, our goal is to investigate the interaction between two pitching hydrofoils that are producing bifurcating wakes instead of the typical reverse von Kármán wakes. In order to ensure that bifurcating flow structures are present, the Strouhal number and aspect ratio are set to St=0.8 and AR=0.5. To determine the effect of this bifurcated wake topology on the flow interactions two different leader-follower arrangements are considered: (1) the follower is in-line and equidistant from the upper and lower branches of the leader’s wake and (2) the upper branch of the leader’s wake is directly impinging on the follower. First, flow measurements were conducted with the use of a tomographic PIV system to detect three-dimensional flow structures in the hydrofoils’ wake. Then, direct force measurements from the follower hydrofoil were obtained for a fixed stream-wise spacing and different cross-stream spacings to quantitatively examine the effect of proximity to the branched wake structures on force generation. Additionally, linear unsteady flow theory was adapted with three-dimensional corrections for comparison with the experimentally obtained thrust and lift forces. This simple theoretical model is used to determine the extent to which flow interactions between two swimmers can be modeled as the superposition of flowfields of two isolated swimmers.

The paper is organized in the following manner. Section 2 details the experimental methodology. Section 3 presents the flow-field measurements, direct force measurements, theoretical model, and comparison with theory. Finally, Section 4 summarizes the main conclusions.

## 2. Experimental Setup and Methods

**Figure 3:**
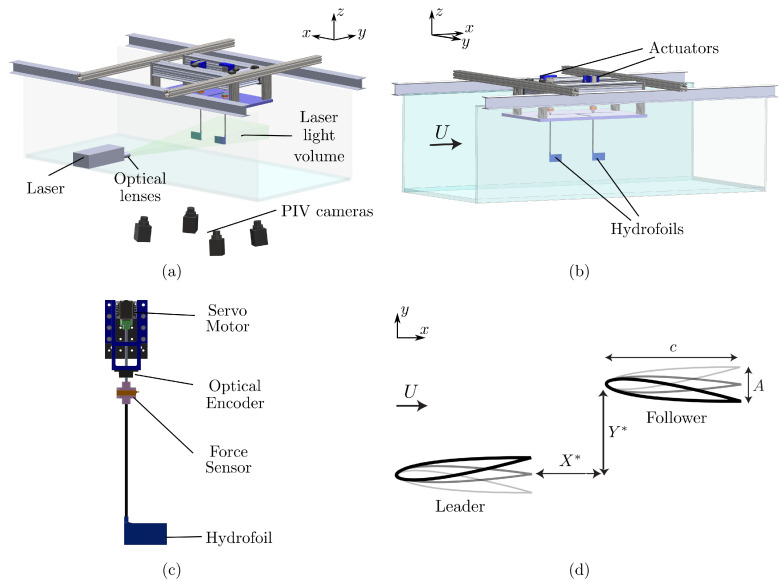
(**a**) A schematic of the tomo PIV experimental facility. (**b**) A schematic of the force measurement facility. (**c**) A detailed schematic of the actuation mechanism. (**d**) A schematic of the interacting hydrofoils’ spatial arrangement showing the streamwise, $X^{*}$, and cross-stream spacing, $Y^{*}$, respectively.

The flow and force measurements were conducted in two different closed loop, free-surface water channels located in the Pennsylvania State University at Berks campus and Lehigh University, respectively. The water channel located at Pennsylvania State University (Figure 3a) has a test section of 3 m length, 0.76 m width, and 0.6 m depth while at Lehigh University (Figure 3b) the test section is of 4.9 m length, 0.93 m width, and 0.61 m depth. The flow speed was constant throughout all the experiments at U=0.046 m/s giving a chord based Reynolds number of Re=4900.

Two identical hydrofoils with a rectangular planform, and NACA 0012 cross-section were designated as the leader and follower (Figure 3d). Each hydrofoil had a chord length of c=0.095 m, and a span length of s=0.0475 m, which gives an aspect ratio of AR=0.5. They were manufactured out of acrylonitrile butadiene styrene (ABS). No deflection (flexing) in the hydrofoil body was inspected during experiments and in flow visualization images, thus, the hydrofoils used in the present study can be characterized as *rigid*. The hydrofoils were actuated about a point 3.2 mm distant from the leading edge with sinusoidal pitching motions by a digital servo motor (Dynamixel MX-64AT), and the motion was tracked by an optical encoder (US Digital E5). A schematic of a single actuator is shown in Figure 3c. The leader was prescribed with a sinusoidal motion defined as $\theta_{L}\left(t\right)=\theta_{0}\sin\left(2\pi ft\right)$ while the follower was actuated similarly, but with a phase difference, $\theta_{F}\left(t\right)=\theta_{0}\sin\left(2\pi ft+\phi \right)$. Here, the pitching frequency denoted with *f*, time with *t*, the pitching amplitude with $\theta_{0}$, and the phase difference or *synchrony* with ϕ. Dimensionless time is the ratio of the time to the period of motion as $t^{*}=t/T=ft$. For the force measurements, the prescribed synchrony between the wings was varied within the range of 0≤ϕ≤2π in increments of π/12 producing 24 synchronies for each wing arrangement. The spatial arrangement of the wings is varied through the manipulation of streamwise and cross-stream spacings, $X^{*}=X/c$ and $Y^{*}=Y/c$, respectively, between each experiment as detailed in Table 1 and shown in Figure 3d. Two different hydrofoil arrangements were considered: (a) an in-line arrangement where the follower is directly downstream of the leader ($X^{*}=0.75$, $Y^{*}=0$), and (b) staggered arrangements where the follower is downstream and off-set in the cross-stream direction ($X^{*}=0.75$, $Y^{*}>0$). The pitching frequency and amplitude were constant throughout all the experiments at f=1.5 Hz and $\theta_{0}=7.5{}^{\circ}$, which gives a Strouhal number of St=fA/U=0.8, and a reduced frequency of k=fc/U=3.1, where $A=2c\sin\theta_{0}$ is the peak-to-peak amplitude of the trailing edge.

An ATI Nano43 six-axis force sensor was used to measure the thrust, and pitching moment acting on the follower wing. The time-varying angular velocity of the wing was calculated from the angular position information recorded from the optical encoder. Then, the total instantaneous power input was calculated from the pitching moment, $M_{\theta }$ and angular velocity, $\dot{\theta }$ as $P_{T}\left(t\right)=M_{\theta }\dot{\theta }$. The inertial power was determined from the same experiments conducted in air, and was subtracted from the total power, $P_{T}\left(t\right)$, to calculate the instantaneous power input to the fluid, P(t). The force measurements were taken for 100 flapping cycles from the follower wing, and each experiment was repeated 10 times. The time-averaged values were calculated for each of these trials, and their mean was calculated to determine the time-averaged thrust, lift, and power. The force data were collected with a National Instrument data acquisition card, and recorded via a MATLAB code by using the National Instrument data acquisition module of MATLAB. The coefficient of net thrust, $C_{T}$, lift, $C_{L}$, and power, $C_{P}$, and the propulsive efficiency, η, for the follower wing are defined as,

(1) $$C_{T}=\frac{\bar{T}}{\frac{1}{2}\rho U^{2}cs},\;C_{L}=\frac{\bar{L}}{\frac{1}{2}\rho U^{2}cs},\;C_{P}=\frac{\bar{P}}{\frac{1}{2}\rho U^{3}cs},\;\eta =\frac{C_{T}}{C_{P}},$$

where ρ is the fluid density. Reported performance variables were normalized with the corresponding isolated wing performance as,

(2) $$\hat{C_{T}}=\frac{C_{T}}{C_{T,\mathrm{iso}}},\;\;\;\hat{C_{P}}=\frac{C_{P}}{C_{P,\mathrm{iso}}},\;\;\;\hat{\eta }=\frac{\eta }{\eta_{\mathrm{iso}}}.$$

Velocity field data were acquired with a Tomo PIV system (LaVision Inc.) consisting of four high-speed cameras (Imager sCMOS) and a 200 mJ pulse Nd:YAG laser (EverGreen 200). A schematic of the cameras and the laser relative to the test section is shown in Figure 3a. The flow was seeded with 20 μm polyamide particles. Optics were used to arrange the thickness of the laser volume so that the entire span of the wing (47.5 mm) was illuminated by the laser. A signal was sent to the programmable timing unit (PTU) at the beginning of each oscillation cycle, triggering all four cameras and the laser. The captured frames were processed by using DaVis10 software. Four different interrogation volume (voxel) sizes were used in the processing of the acquired images with starting voxel size of 96×96×96 with 75% overlap, and final voxel size of 48×48×48 with 75% overlap. Twenty-four discrete phases across a pitching cycle were acquired by averaging each phase over 50 oscillation cycles. Time-averaged data was obtained by averaging these phases distributed equally over one oscillation cycle.

## 3. Results

### 3.1. Isolated Hydrofoil Performance and Wake Measurements

The net thrust, power, and lift coefficients, and propulsive efficiency for an isolated hydrofoil are reported in Table 2. The high Strouhal number and low aspect ratio of the hydrofoil lead to moderate thrust production, high power and low efficiency.

**Figure 4:**
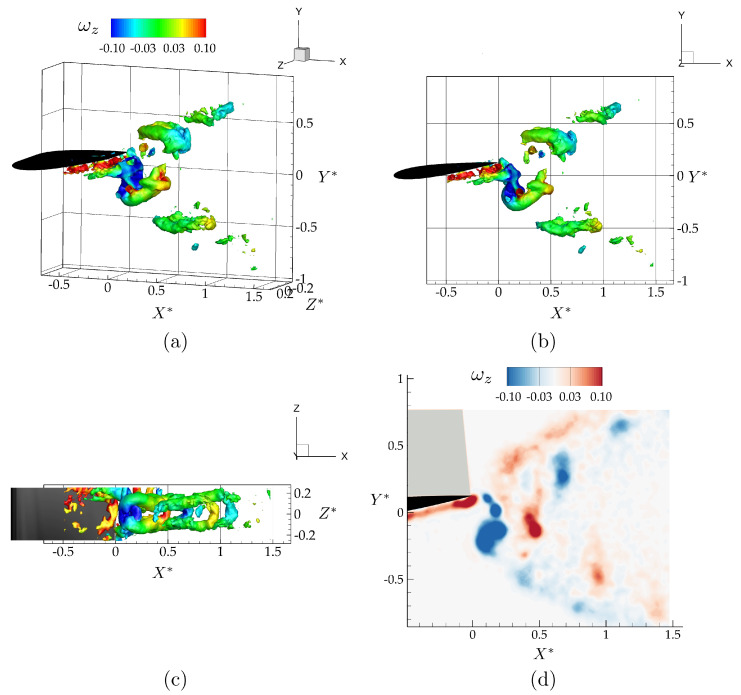
Wake structures shedding from the trailing edge of an isolated wing shown with $\lambda_{2}=-0.07$ isocontours at the dimensionless time of $t^{*}=0.25$, as an isometric (**a**), side (**b**), and top (**c**) view of the flowfield, and vorticity contours ($\omega_{z}$) in the mid-span plane (**d**).

Figure 4 shows isometric, side, and bottom views of the three-dimensional vortex structures, and the spanwise vorticity field, $\omega_{z}$, at the mid-span of the isolated hydrofoil. The vortex structures in Figure 4a–c are identified using the $\lambda_{2}$ criterion, with $\lambda_{2}=-0.07$ and are colored with corresponding values of spanwise vorticity. The $\lambda_{2}$ criterion detects pressure minima in a plane after eliminating unsteady staining and viscous effects [40]. It is defined as the median of three eigenvalues of $S^{2}+\Omega^{2}$, where *S* is the rate-of-strain tensor, and Ω is the rate-of-rotation tensor. The three corresponding eigenvalues, $\lambda_{1}$, $\lambda_{2}$, and $\lambda_{3}$, are ordered in such a way that $\lambda_{1}\geq \lambda_{2}\geq \lambda_{3}$. A point in the velocity field is a part of a vortex core only if the second eigenvalue is negative, $\lambda_{2}<0$.

As anticipated, the vortex structures form a bifurcating wake consisting of two vortex rings per oscillation cycle that propagate downstream and away from the $Y^{*}=0$ plane. This type of bifurcating wake has been reported in previous studies of low-aspect ratio flat plate experiments [36] and simulations [39], and of low-aspect ratio ellipsoidal wing simulations [37].

**Figure 5:**
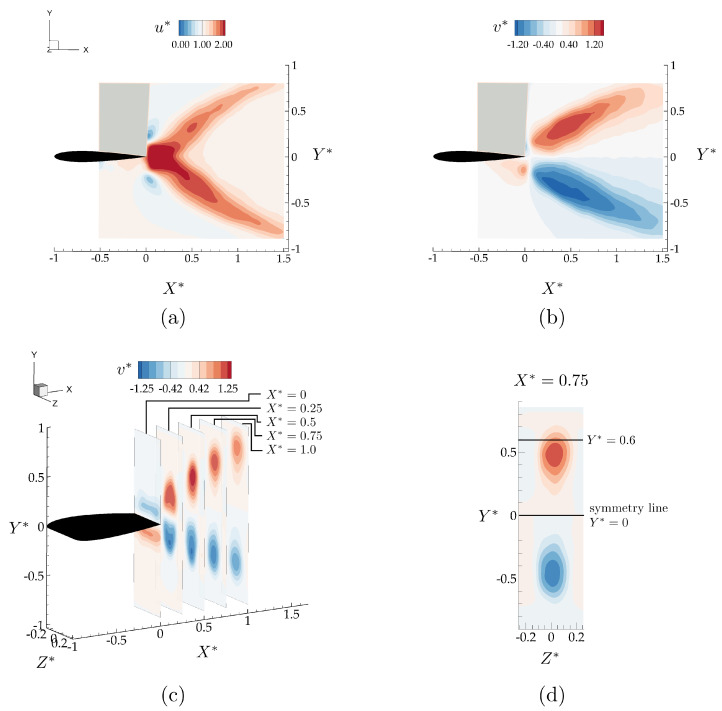
Time averaged velocity field components, (u,v), normalized with the free-stream velocity, *U*, for the isolated wing in the mid-span plane (**a**,**b**), and different YZ planes (**c**,**d**).

Figure 5 shows the *x*- and *y*-components of the time-averaged velocity field normalized with the free-stream velocity, $u^{*}=u/U$, and $v^{*}=v/U$, respectively. In the time-average, propagating vortex rings create a bifurcating momentum jet with upper and lower branches directing momentum upward and downward, respectively. Figure 5b shows that for $Y^{*}>0$ there is up-wash in the time average, while for $Y^{*}<0$ there is down-wash in the time average with the magnitude of *y*-component of the velocity increasing by 25% above the free-stream velocity in both directions. Figure 5c shows *Y*-*Z* slices of time-averaged $v^{*}$ from $0\leq X^{*}\leq 1$. The accelerated region in the wake becomes stretched in the *y*-direction and compressed in the *z*-direction with respect to the accelerated regions at the trailing edge. At $X^{*}=1$ the accelerated region spans close to half the span-length of the hydrofoil. There is a slight asymmetry in the wake evident from the time-averaged velocity fields in Figure 5a,d, which causes the downwash region to be 0.05 chord closer to the symmetry line. The non-zero lift generation from the isolated wing can be attributed to this asymmetry in the wake.

### 3.2. Wake Measurements of Two Interacting Hydrofoils

Here, the flowfields are presented for an in-line arrangement with $X^{*}=0.75$ and $Y^{*}=0$ and a staggered arrangement with $X^{*}=0.75$ and $Y^{*}=0.6$. These two cases were selected to examine two different interaction modes with the bifurcated wake based on the isolated case (see Figure 5a–d). In Figure 5c, the YZ planes in the isolated hydrofoil wake shows that $X^{*}=0.75$ plane provides enough cross-stream spacing between the accelerated flow branches to have these two distinct interaction modes. In $X^{*}=0.75$ plane, direct wake impingement on the follower is expected if the hydrofoil is to be located within one of the accelerated flow regions in a staggered arrangement ($Y^{*}=0.6$), as opposed to an inline arrangement where the follower is located on the symmetry line ($Y^{*}=0$) between these accelerated branches with the time-averaged velocity magnitude minimally altered from the free-stream velocity, *U* (see Figure 5d).

**Figure 6:**
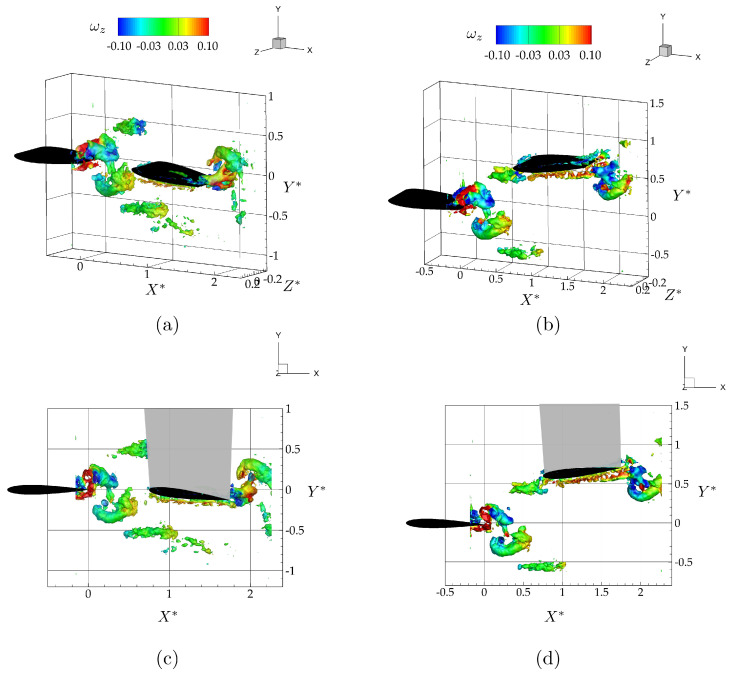
Isometric (top row; **a**, **b**) and side view (bottom row; **c**, **d**) of three-dimensional vortex structures for the in-line (left column; **a**, **c**) and the staggered case (right column; **b**, **d**) shown at the time instant, $t^{*}=1$. The vortex structures are defined by the isosurface $\lambda_{2}=-0.07$ and are colored with corresponding values of spanwise vorticity.

Figure 6a,c show isometric and side views of the wake as visualized with $\lambda_{2}=-0.07$ isocontours colored with span-wise vorticity for the leader and follower hydrofoils oscillating with a synchrony of ϕ=π/2 in an in-line arrangement. As expected from the isolated hydrofoil data, there is no direct impingement of the vortex rings shedding from the leader with the follower. The two counter-rotating vortex rings shedding into the wake during each oscillation cycle pass above and below the follower. Figure 6b,d show isometric and side views of the wake for the hydrofoils oscillating with a synchrony of ϕ=3π/2 in a staggered arrangement. As predicted by the isolated hydrofoil data, there is a direct impingement of the upper branch of the leader’s shed vortex rings onto the leading edge of the follower.

**Figure 7:**
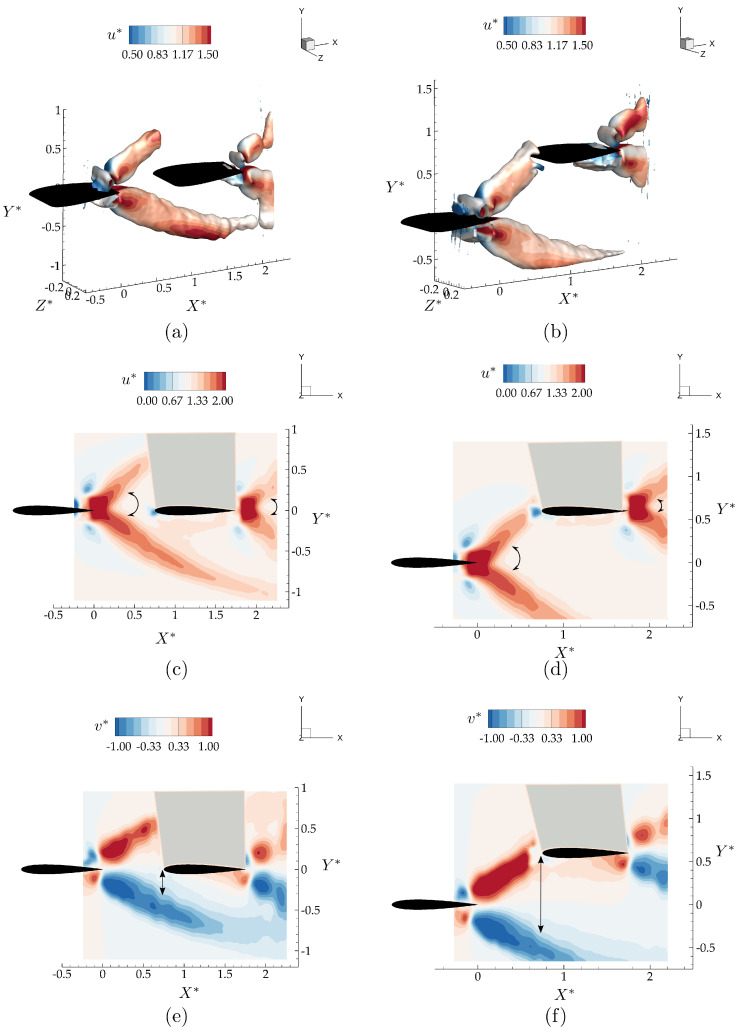
Time-averaged velocity for the in-line arrangement (left column; **a**, **c**, **e**) and the staggered arrangement (right column; **b**, **d**, **f**). The top row (**a**, **b**) presents the $|v^{*}|=0.5$ isocontour colored with their corresponding time-averaged velocity, $u^{*}$. The middle row (**c**, **d**) presents the time-averaged streamwise velocity at the mid-span plane, while the bottom row (**e**, **f**) presents the time-averaged cross-stream velocity at the mid-span. Arcs and lines with arrows show the expansion of the streamwise accelerated flow branches, and the distance between the leading edge of the follower and the time-averaged downwash region, respectively.

Figure 7 presents $|v^{*}|=0.5$ isocontours colored with their corresponding time-averaged $u^{*}$ values along with mid-span slices of $u^{*}$ and $v^{*}$ for the in-line and staggered arrangements of the leader and follower. For the in-line arrangement, although the momentum jet expansion stays similar between the hydrofoils indicated with arrow arcs in Figure 7, there is an expanded region of streamwise accelerated flow over the lower surface of the follower. Additionally, a time-averaged downwash of $v^{*}=0.3$ extends to the leading edge of the follower due to the small distance between the follower and the accelerated flow region indicated with black arrows in Figure 7e. Apart from these small changes in the flow-field, surprisingly, the presence of the follower seems like a simple superposition of the flow states for an isolated leader and follower.

In the staggered arrangement, the leader’s upper branch momentum jet is directly impinging on the follower, giving rise to the greatest potential for nonlinear interactions, that is, for superposition arguments to fail. The presence of the follower is observed to slightly change the direction of the upper branch jet, thereby expanding the bifurcating wake of the leader as shown in Figure 7b,d. This is observed to increase the cross-stream component of the velocity in the core of the upper branch jet. Compared to the in-line case, in Figure 7d, the region with accelerated $u^{*}$ around the lower surface of the wing is much smaller for this staggered interaction case as presented. However, Figure 7f shows a large upwash region formed up on the lower surface of the follower inducing a cross-stream velocity in the time-average of $v^{*}=0.4$. In the direct impingement case, there are more nonlinear interactions observed, yet, as in the in-line case, these alterations to the flow-field remain to be minor.

These findings motivate us to consider modeling schooling interactions as the superposition of the flow state for the leader and follower in isolation. Can classic linear unsteady flow theory for an isolated pitching and heaving plate capture the major schooling interactions between two hydrofoils in terms of thrust, lift, and efficiency?

### 3.3. Linear Unsteady Hydrofoil Theory

To model these interactions, one can use classic inviscid gust response theories as a superposition of a pitching hydrofoil (follower) in an unsteady gust field (wake of the leader). Amongst the potential flow solutions, such as Wagner [41] and von Kármán & Sears [42], a simpler gust model based on Theodorsen’s [43] and Garrick’s work [44] can be proposed. Garrick advanced Theodorsen’s theory by accounting for the singularity in the vorticity distribution at the leading edge to solve for the thrust generation from an airfoil or hydrofoil undergoing heaving, pitching, and/or flap motions. Garrick’s solution assumes potential flow over: (a) a two-dimensional, (b) infinitesimally-thin hydrofoil, (c) undergoing small amplitude, (d) harmonic motions with (e) a non-deforming, planar wake behind the hydrofoil. Moreover, since this is an inviscid flow solution, induced shedding of vorticity at the leading edge from interaction with the gust field is not accounted for, even though it is known to exist in schooling interactions [12,19]. In the present study, we will compare direct force measurements of the follower hydrofoil to the quasi-steady Garrick solution with three-dimensional corrections. This theory already provides a solution for the thrust, unlike the other classic gust theories. In this way, we will be able to examine whether a simple theory can approximate the basic flow interactions that occur during schooling.

We utilize Garrick’s theory to determine the forces acting on the follower. First, the theory is taken in the quasi-steady approximation where the influence of the wake shed from the follower hydrofoil is neglected, that is, Theodorsen’s lift deficiency function C(k)=1. This approximation relaxes the assumption that the hydrofoil motion must be harmonic and it can undergo arbitrary motions [45]. Following the potential flow solution from Theodorsen [43] and Garrick [44], the lift and thrust forces can be decomposed into their non-circulatory (added mass) and circulatory components, and expressed as,

(3) $$\begin{array}{c} L_{2D}=\underset{L_{NC}}{\underbrace{-\frac{1}{4}\rho c^{2}s\left(U\pi \dot{\alpha }+\pi \ddot{h}+\frac{\pi }{2}c\ddot{\alpha }\right)}}+\underset{L_{C}}{\underbrace{-\pi \rho UcsQ}}\\ T_{2D}=\underset{T_{NC}}{\underbrace{-\frac{1}{4}\rho c^{2}s\left(U\pi \dot{\alpha }\alpha +\pi \ddot{h}\alpha +\frac{\pi }{2}c\ddot{\alpha }\alpha \right)}}+\underset{T_{C}}{\underbrace{\frac{\pi }{2}\rho csS^{2}-\pi \rho UcsQ\alpha }}\\ \;\mathrm{where}\;S=\frac{1}{2\sqrt{2}}\left(4Q-c\dot{\alpha }\right),\\ \;\mathrm{and}\;Q=U\alpha +\dot{h}+\frac{3}{4}c\dot{\alpha }. \end{array}$$

Here, the subscript ${\left(.\right)}_{NC}$ and ${\left(.\right)}_{C}$ denote non-circulatory and circulatory forces, respectively. The pitching angle, α, pitching rate, $\dot{\alpha }$, and angular acceleration, $\ddot{\alpha }$, are input from the prescribed pitching motion of the follower about the leading edge. The heaving velocity, $\dot{h}$, and heaving acceleration, $\ddot{h}$, are input from the *v*-component of the velocity field induced by the leader, which can be considered as a time-varying upwash/downwash.

**Figure 8:**
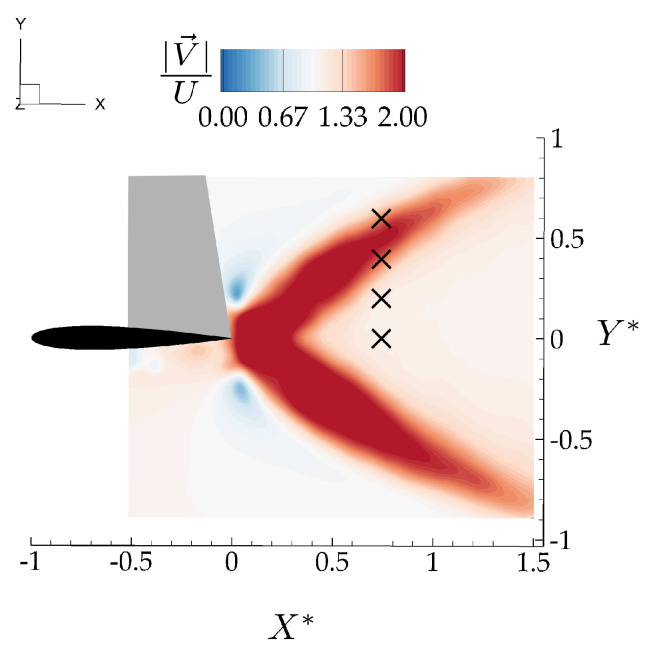
Locations of the leading edge of the follower foil (*x* markers) relative to the time-averaged velocity jets from the flowfield of the isolated leader.

The induced “gust” velocity field is extracted directly from PIV data of the *isolated leader*, that is, different from the flowfield produced when two hydrofoils are interacting. The vertical component of the isolated leader velocity field, *v*, varies over 24 district phases equally distributed over an oscillation cycle. For each phase, vertical velocity information at locations corresponding to the leading edge location of the follower, $X^{*}=0$, and $0\leq Y^{*}\leq 0.6$, were extracted from 50 different locations equally distributed in the spanwise direction over one span-length. The vertical velocity was then averaged over the spanwise direction to represent a characteristic time-varying vertical velocity for each $\left(X^{*},Y^{*}\right)$ case. The $\left(X^{*},Y^{*}\right)$ locations relative to the time-averaged jet structures are shown in Figure 8.

Since the interacting hydrofoils are of low aspect ratio (AR=0.5), three-dimensional corrections are introduced to modify the classic two-dimensional quasi-steady theory. Following [46,47] the added mass forces are corrected by a factor of AR/(AR+1), while the circulatory forces are corrected by Helmbold’s factor of $A\;R/[\sqrt{{A\;R}^{2}+4}+2]$, which is for low aspect ratio foils in the range of 0.5≤AR≤6 [48,49,50]. The corrected thrust and lift are,

(4) $$L=L_{NC}\frac{A\;R}{A\;R+1}+L_{C}\frac{A\;R}{\sqrt{{A\;R}^{2}+4}+2}$$

(5) $$T=T_{NC}\frac{A\;R}{A\;R+1}+T_{C}\frac{A\;R}{\sqrt{{A\;R}^{2}+4}+2}-\frac{1}{2}C_{D,\mathrm{iso}}\rho u^{2}cs$$

Note that the thrust model has a drag term where the drag coefficient comes from the isolated hydrofoil measurements, and *u* is the streamwise velocity at locations corresponding to the leading edge of the follower hydrofoil in the isolated hydrofoil wake as cross-marked in Figure 8, and averaged in the spanwise direction over one span length.

### 3.4. Follower Performance Comparison with Theory

**Figure 9:**
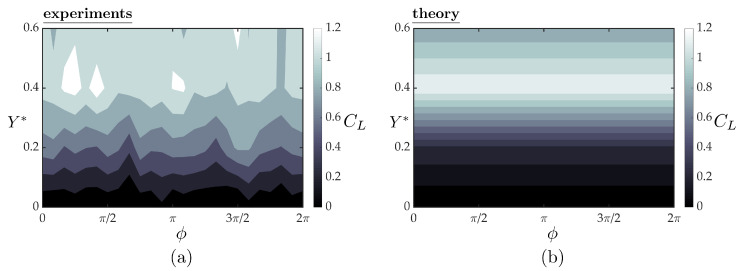
Lift of the follower hydrofoil as a function of the synchrony and cross-stream spacing from the experiments (**a**) and the three-dimensional quasi-steady theory (**b**). The follower is at a fixed streamwise spacing of $X^{*}=0.75$ for all the cases presented here.

Figure 9a,b present the mean lift coefficients from the experiments and theory, respectively, as functions of synchrony and cross-stream spacing for a fixed streamwise spacing of $X^{*}=0.75$. At a fixed synchrony, the lift increases with increasing cross-stream spacing until a peak lift coefficient of $C_{L}=1.2$ is reached around $Y^{*}=0.4$, and then it decreases with further increase in cross-stream spacing for $Y^{*}>0.4$. This trend in the lift with increasing cross-stream spacing and the peak lift coefficient are well-predicted by the simple theory. By inspecting the theoretical solution, the trend in the lift coefficient is driven by the time-averaged induced angle of attack of the upper branch of the bifurcating jet. In fact, steady thin airfoil theory with the three-dimensional corrections discussed above drive the lift response. This is reflected in the theoretical solution with the lack of a variation in lift with variation in the synchrony. In the experiments there is slight variation in the lift response as a function of the synchrony that is not accounted for in the simple theory.

**Figure 10:**
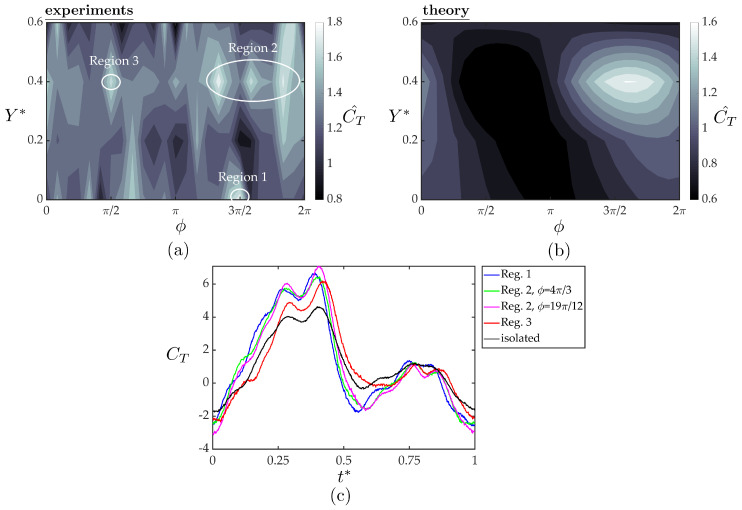
Normalized thrust coefficients of the follower hydrofoil acquired from the experiments (**a**) and the theoretical solution (**b**) as a function of cross stream spacing and synchrony. Three high thrust regions are denoted on the experimental data with white circles. The temporal variation of thrust coefficient within one oscillation cycle for the marked peak thrust cases compared to isolated foil case are given in (**c**). For each case, instantaneous thrust forces were phase averaged over 100 oscillation cycles.

Figure 10a,b present the normalized thrust coefficients of the follower from the experiments and theory as functions of the synchrony and cross-stream spacing. The experimental data exhibit high variability of the normalized thrust with variation in both the synchrony and cross-stream spacing. There are numerous peaks in normalized thrust, which can be grouped into three regions (see Figure 10a). Region one shows a single thrust peak at $Y^{*}=0$ and ϕ=3π/2 with a thrust increase of 80% over the isolated hydrofoil. Region two is a grouping of three thrust peaks centered at $Y^{*}=0.4$ and ϕ=19π/12 (≈3π/2) representing a 67–78% increase in thrust over the isolated hydrofoil. Region three shows a single peak centered at $Y^{*}=0.4$ and ϕ=π/2 representing a 67% increase in thrust over the isolated hydrofoil. The theoretical solution shows much less variability than the experimental data. It predicts a peak thrust region centered at $Y^{*}=0.4$ and ϕ=19π/12 (same as region two) and a 61% increase in thrust over the isolated hydrofoil. Figure 10c presents the temporal variations of the thrust coefficient within one oscillation cycle plotted as an average over 100 oscillation cycles for the peak thrust regions observed in the measured data in comparison with the isolated hydrofoil. As expected, all the cases examined here shows two peaks in instantaneous thrust production. Although all the cases follow similar trends around the second peak, the higher first peaks in the instantaneous data for the interaction cases than of the isolated foil are found to be the lead cause of the reported thrust gains for the follower hydrofoil compared to a hydrofoil in isolation (Figure 10a).

The location and thrust increase predicted by the simple theory is surprisingly similar to the experimental data in region two. However, the theory does not capture the largest peak thrust in region one nor the small peak in region three. The discrepancy between the theory and experiments indicates that either the spatial variation in the wake flow impinging on the follower or nonlinearities in the flow such as induced separation on the follower gives rise to the observed variability in the follower thrust; neither of which are accounted for in the simple theory. The theory uses the cross-stream velocity extracted from the isolated wake to predict thrust production for an hydrofoil interacting with the isolated hydrofoil wake. This velocity field data does not contain any information about the small alterations in the flowfield due to the presence of the follower hydrofoil, such as, accelerated time-averaged streamwise flow, or a large upwash region inducing non-zero cross-stream velocity in the time-average over the lower surface of the follower hydrofoil, in an in-line and a staggered arrangement, respectively, as discussed in Section 3.2. These small alterations can potentially change both instantaneous and time-averaged thrust production and may be the cause of the thrust peaks in the experimental data that could not be captured with the simple theory. For future work, a more advanced theory based in the work of von Kármán & Sears [42] could account for the spatial variation in the wake flow acting on the follower and isolate the effect of flow nonlinearities, i.e., flow structures that are not the superposition of the flowfields of two isolated hydrofoils.

**Figure 11:**
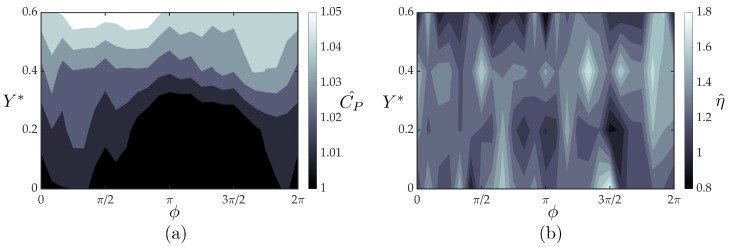
Measured normalized (**a**) power coefficient and (**b**) efficiency for the follower hydrofoil.

Figure 11a,b present the measured normalized follower power and the normalized follower efficiency, respectively, as a function of the synchrony and cross-stream spacing. The measured power exhibits little variation (1–5%) from the isolated case, leading to nearly identical trends between normalized thrust and efficiency with three regions of peak efficiencies ranging from a 63–81% increase in efficiency over the isolated hydrofoil. The minute variation in power suggests that introducing a theoretical model for the power consumption is not needed in this case to capture the efficiency trends.

## 4. Conclusions

We have presented experiments for two interacting hydrofoils of AR=0.5 in in-line and staggered arrangements, as they shed a branched wake with two vortex rings per oscillation cycle bifurcating in opposite cross-stream directions. In the in-line arrangement ($Y^{*}=0$), the follower is equidistant from the wake branches and there is no direct impingement of the leader’s wake on the follower. In contrast, in the staggered arrangement ($Y^{*}=0.6$), there is a direct vortex impingement onto the follower’s leading edge. Even during direct impingement in the staggered arrangement only minor alterations to the leader’s wake are observed suggesting that the flowfield produced by the two interacting hydrofoils may be modeled as the superposition of flowfields produced by two hydrofoils in isolation. Motivated by this observation, Garrick’s linear theory was adapted to examine the change in lift and thrust forces generated by the follower as it interacts with the leader’s wake. The model was corrected for three-dimensional effects, and compared with direct force measurements. Both linear theory predictions and experiments show a peak in lift when the follower is located close to the upper wake branch at $Y^{*}=0.4$. This trend occurs due to the mean effective angles of attack induced in the branches of the bifurcated wake structure. The linear theory solution predicts a peak thrust region centered at $Y^{*}=0.4$ and ϕ=19π/12 where the follower produces 61% more thrust than in isolation. The experimental data shows that this region is in fact split into three thrust peaks where the follower produces 67–78% more thrust than in isolation, in good agreement with the theory. However, the follower is found to produce 80% more thrust than in isolation at $Y^{*}=0$ and ϕ=3π/2 and 67% more thrust at $Y^{*}=0.4$ and ϕ=π/2, neither of which is predicted by the simple linear theory. These additional peaks in thrust are likely driven by spatial variations in the flowfield acting on the follower or nonlinear flow interactions neither of which are accounted for in the theory. The power coefficient shows negligible variations as the synchrony and cross-stream spacing are varied such that it is within 5% of the power for a hydrofoil in isolation. Consequently, the normalized efficiency shows nearly identical peaks as the normalized thrust with a 63-81% increase in efficiency over the isolated hydrofoil.

Overall, the results of this study indicate that unsteady linear potential flow theory can provide a foundation to understand and model flow interactions that occur in schools of fish. Moreover, the results suggest that eels may experience a thrust and efficiency increase of 63–81% when schooling in either an in-line arrangement where a follower is swimming between two momentum jet branches of a leader or in a staggered arrangement where one of the branched momentum jets of the leader directly impinges on the follower.

## Figures and Tables

**Table 1:** Experimental parameters and input variables used in the present study.

$X^{*}$	0.75	
$Y^{*}$	0–0.6	0.2 increments
ϕ	0–2π	π/12 increments
*k*	3.1	
St	0.8	
$\theta_{0}$	7.5°	(A/c=0.26)
*f*	1.5	

**Table 2:** Time-averaged net thrust, power, lift and drag coefficients, as well as propulsive efficiency of an isolated hydrofoil at St=0.8 and k=3.1. ±(·) represents the standard deviation calculated from 10 experimental trials.

Coefficients	
$C_{T,\mathrm{iso}}$	0.47±0.13
$C_{P,\mathrm{iso}}$	7.65±0.11
$C_{L,\mathrm{iso}}$	0.26±0.11
$C_{D,\mathrm{iso}}$	0.43±0.04
$\eta_{,\mathrm{iso}}$	0.06±0.01
